# Supplementary material for: Structural mechanism of lipid modulation of pentameric ligand-gated ion channel activity
Source: bioRxiv. 2025 Oct 7:2025.10.07.680764. Preprint. [Version 1] doi: 10.1101/2025.10.07.680764 (PMC12632492; doi:10.1101/2025.10.07.680764)
Supplement: Supplement 3 [file NIHPP2025.10.07.680764v1-supplement-3.pdf]

bioRxiv preprint doi: https://doi.org/10.1101/2025.10.07.680764; this version posted October 7, 2025. The copyright holder for this preprint (which was not certified by peer review) is the author/funder, who has granted bioRxiv a license to display the preprint in perpetuity. It is made available under aCC-BY 4.0 International license.

Supplementary Figure 1

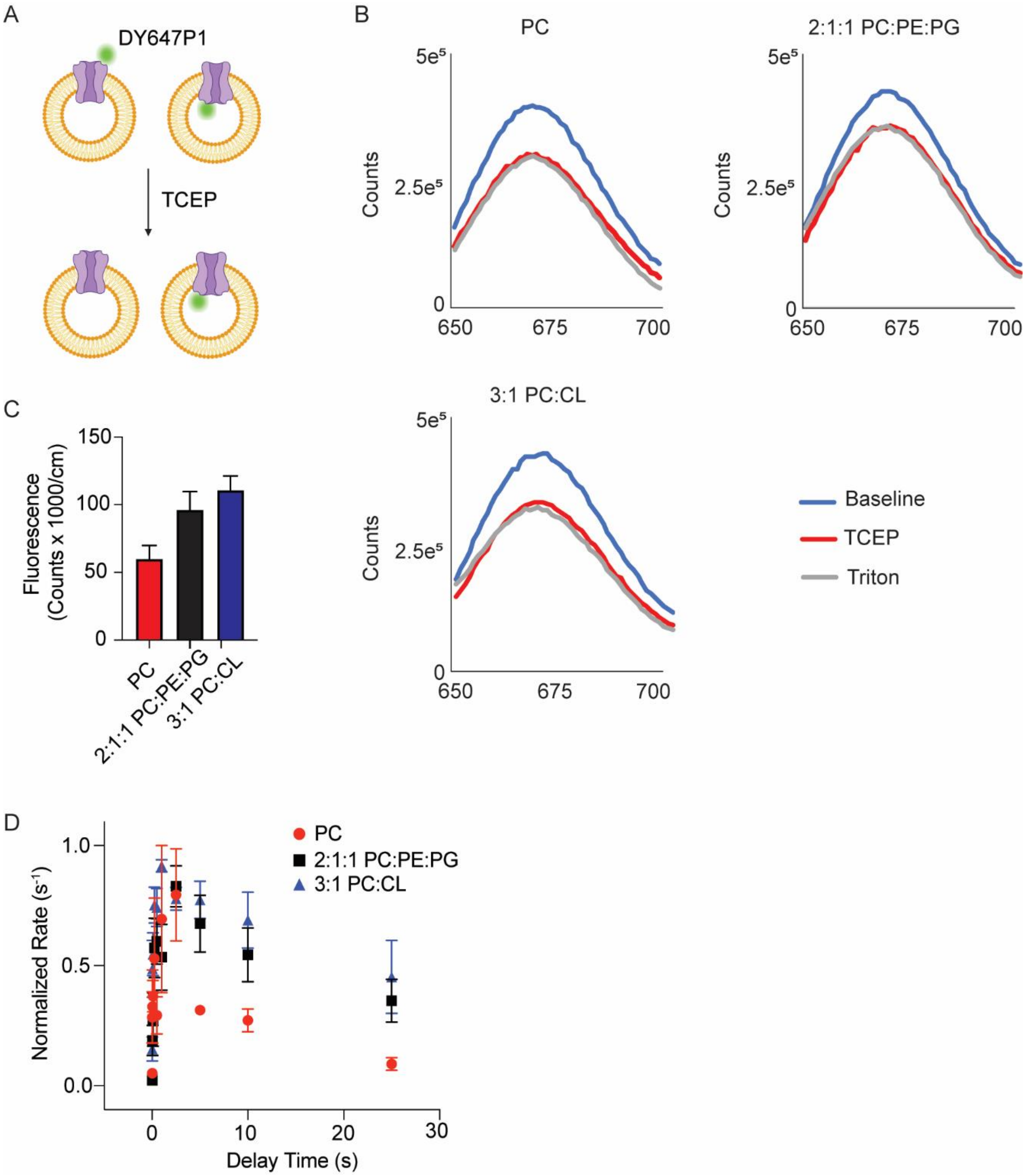

Supplementary Figure 1: Fluorescence and Activity of DY647P1-labeled ELIC (A6C/C300S/C313S) in liposomes. (A) Schematic of ELIC (A6C/C300S/C313S) labeled with DY647P1 on the cysteine in position 6 and reconstituted in liposomes in both orientations. Treatment with TCEP only quenches those channels with the ECD facing the extra-liposomal space. (B) Fluorescence spectra of DY647P1-labeled ELIC (A6C/C300S/C313S) in liposomes with POPC, 2:1:1 POPC:POPE:POPG, and 3:1 POPC:DPOCL

bioRxiv preprint doi: https://doi.org/10.1101/2025.10.07.680764; this version posted October 7, 2025. The copyright holder for this preprint (which was not certified by peer review) is the author/funder, who has granted bioRxiv a license to display the preprint in perpetuity. It is made available under aCC-BY 4.0 International license.

before TCEP, after TCEP, and after Triton X-100. The data indicate that most channels have the ECD facing the extra-liposomal space. The residual fluorescence after Triton X-100 treatment indicates that some fluorescently-labeled ELIC is not accessible to TCEP possibly because the protein is aggregated and not reconstituted in liposomes. To optimize the fluorescent signal, DY647P1-labeled ELIC (A6C/C300S/C313S) was reconstituted at 3 μg protein per 1 milligram lipid for this experiment. (C) The difference in fluorescence signal of DY647P1-labeled ELIC (A6C/C300S/C313S) before and after TCEP, indicating the quantity of ELIC with the ECD facing the extra-liposomal space (n = 3). (D) Tl^+ flux rates of DY647P1-labeled ELIC (A6C/C300S/C313S) reconstituted in liposomes with POPC, 2:1:1 POPC:POPE:POPG, and 3:1 POPC:DPOCL. The data are normalized to the peak rate (n = 3-8, data from Figure 1B). All data are shown as mean ± SEM for (n) independent experiments.

bioRxiv preprint doi: https://doi.org/10.1101/2025.10.07.680764; this version posted October 7, 2025. The copyright holder for this preprint (which was not certified by peer review) is the author/funder, who has granted bioRxiv a license to display the preprint in perpetuity. It is made available under aCC-BY 4.0 International license.

Supplementary Figure 2

A

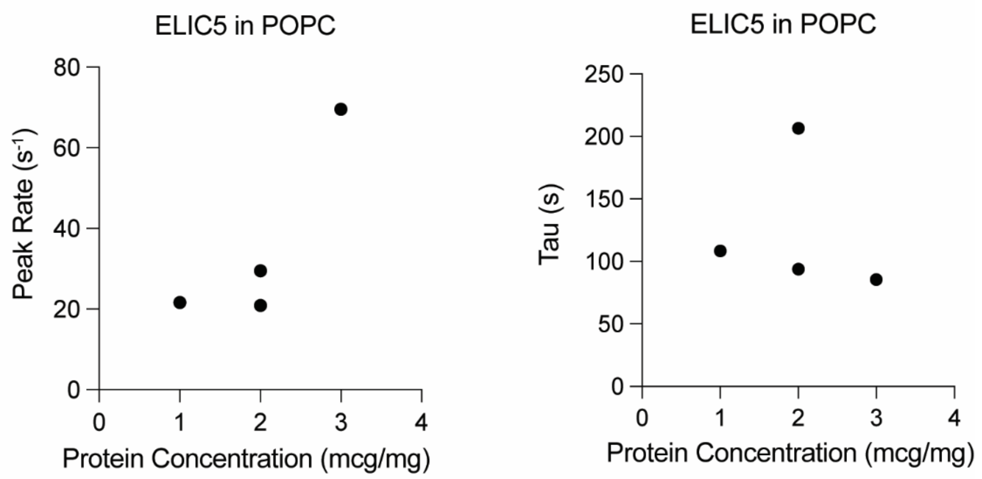

B

Condition	n	EC_{50} (mM)	A (s^{-1})
**PC**	1.26 +/- 0.26	0.18 +/- 0.10	11.7 +/- 1.4
**3:1 PC:PE**	5.36 +/- 0.69	0.12 +/- 0.02	316.0 +/- 11.6
**2:1:1 PC:PE:PG**	4.25 +/- 0.65	0.15 +/- 0.03	376.5 +/- 19.5
**3:1 PC:CL**	4.47 +/- 0.55	0.15 +/- 0.03	319.3 +/- 22.1

Supplementary Figure 2: Relationship between ELIC5 peak agonist response or activation rate and the quantity of protein added to the liposome preparations. (A) To examine whether ELIC5 peak response or activation rate in POPC liposomes is dependent on the amount of protein used, ELIC5 activation was examined as in Figure 1C using different amounts of ELIC5 (micrograms of ELIC5 per milligram of lipid) for the liposome reconstitution. Shown are the peak Tl^+ flux rates (*left*) and the time constants of activation (*right*) as a function of ELIC5 protein concentration. As expected, the peak agonist response increases with protein concentration but not the time constant of ELIC5 activation. (B) Results of fitting the Hill equation to the data in Figure 1D (n = 3-6, ± SD). n is the Hill coefficient, EC_{50} is the concentration of propylamine producing half maximal response, and A is the maximum activity (Tl^+ flux rate).

bioRxiv preprint doi: https://doi.org/10.1101/2025.10.07.680764; this version posted October 7, 2025. The copyright holder for this preprint (which was not certified by peer review) is the author/funder, who has granted bioRxiv a license to display the preprint in perpetuity. It is made available under aCC-BY 4.0 International license.

Supplementary Figure 3

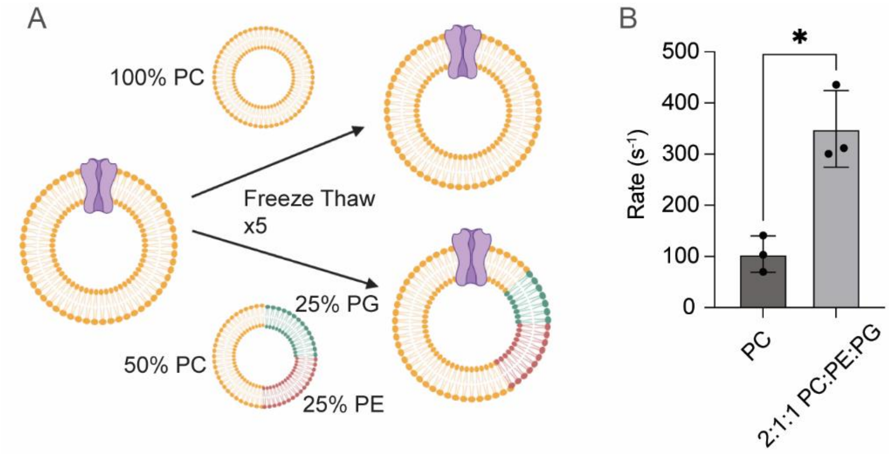

Supplementary Figure 3: ELIC5 activity can be rescued after reconstitution in POPC liposomes. (A) Schematic of the experiment where ELIC5 is reconstituted in POPC liposomes. The preparation is split and mixed with equal quantities of POPC or 2:1:1 POPC:POPE:POPG liposomes. Each sample was then treated with 5 rounds of freeze-thaw. Due to liposome fusion, the anticipated result is ELIC5 proteliposomes with POPC-only or up to 12.5% POPE and 12.5% POPG. (B) Tl^+ flux rates of ELIC5 from samples treated by freeze thaw with POPC liposomes or 2:1:1 POPC:POPE:POPG liposomes (n = 3). All data are shown as mean ± SEM for (n) independent experiments. * $p<0.01$ from paired T-test. To ensure adequate Tl^+ flux rates, 3 micrograms of ELIC5 per milligram of lipid was used for each preparation. Unless otherwise specified, all other stopped-flow thallium flux experiments in this study used 1 microgram of ELIC5 per milligram of lipid.

bioRxiv preprint doi: https://doi.org/10.1101/2025.10.07.680764; this version posted October 7, 2025. The copyright holder for this preprint (which was not certified by peer review) is the author/funder, who has granted bioRxiv a license to display the preprint in perpetuity. It is made available under aCC-BY 4.0 International license.

Supplementary Fig. 4

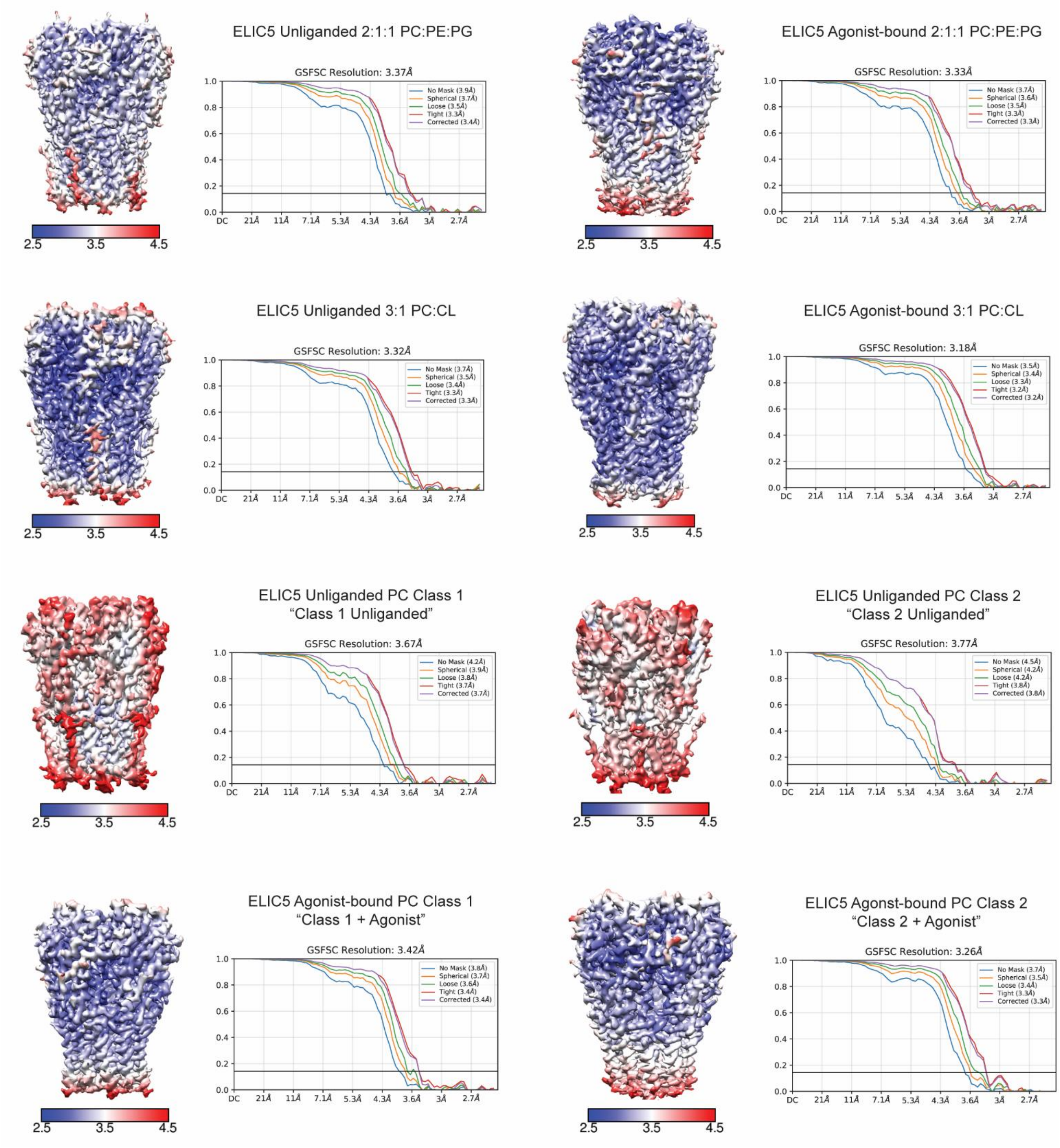

Supplementary Figure 4: Local resolution and FSC curves for cryo-EM structures. For each structure, on the left is the cryo-EM map colored according to local resolution and on the right is the FSC curve from cryoSPARC.

bioRxiv preprint doi: https://doi.org/10.1101/2025.10.07.680764; this version posted October 7, 2025. The copyright holder for this preprint (which was not certified by peer review) is the author/funder, who has granted bioRxiv a license to display the preprint in perpetuity. It is made available under aCC-BY 4.0 International license.

Supplementary Figure 5

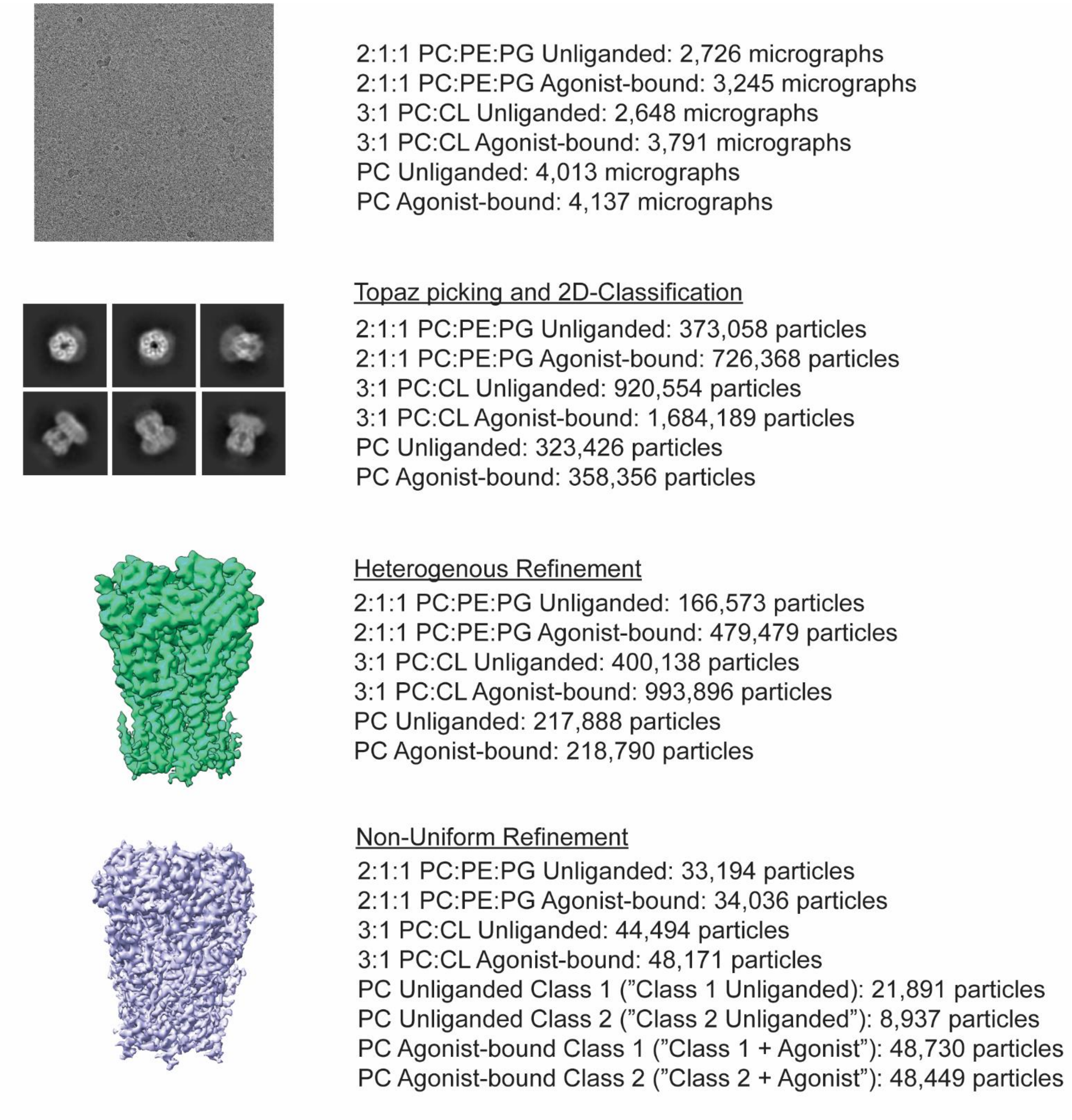

Supplementary Figure 5: Summary of the single particle cryo-EM analysis in cryoSPARC. Listed are the numbers of micrographs and particles included in each step of the analysis including particle picking, heterogeneous refinement and non-uniform refinement. In the case of the POPC structures, classes 1 and 2 are structures from 3DVA clustering.

bioRxiv preprint doi: https://doi.org/10.1101/2025.10.07.680764; this version posted October 7, 2025. The copyright holder for this preprint (which was not certified by peer review) is the author/funder, who has granted bioRxiv a license to display the preprint in perpetuity. It is made available under aCC-BY 4.0 International license.

Supplementary Figure 6

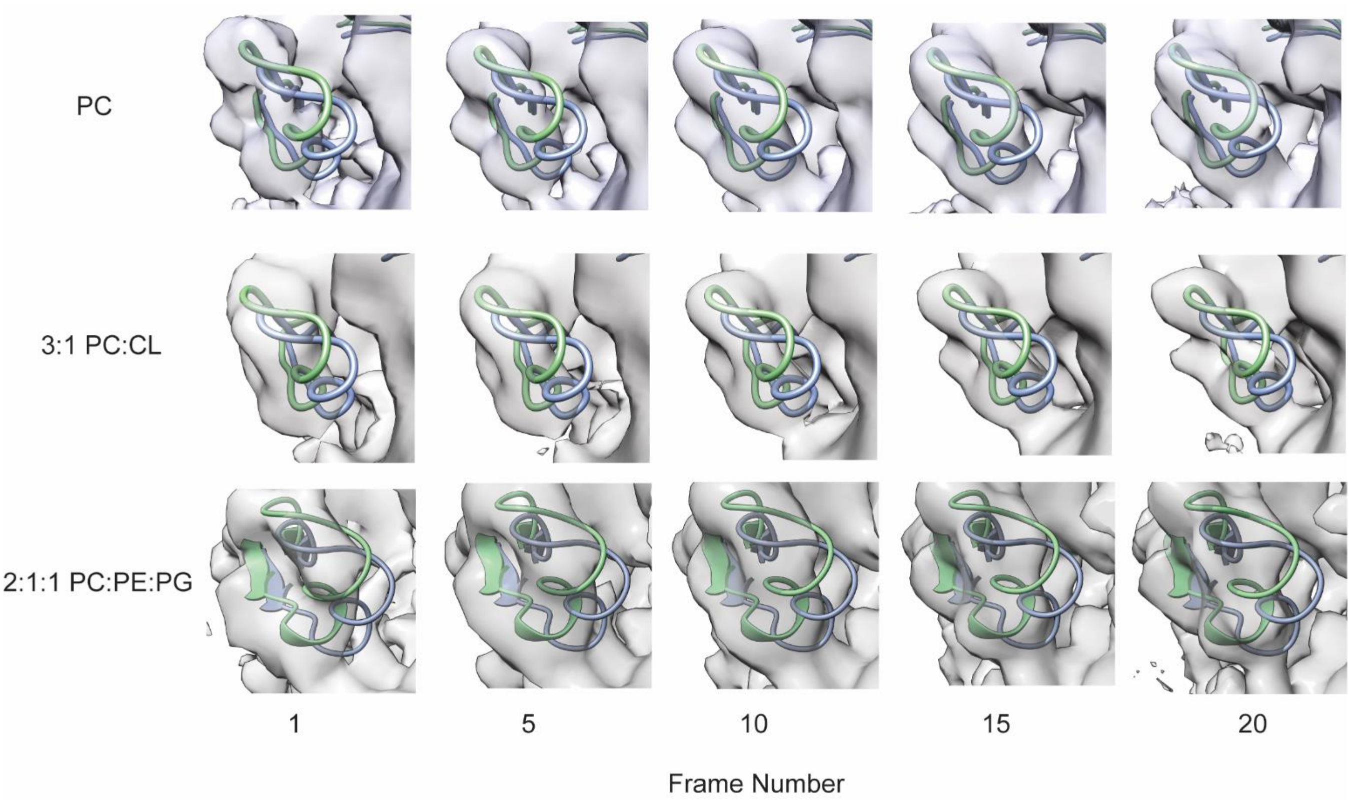

Supplementary Figure 6: Cryo-EM maps from 3DVA snapshots from ELIC5 unliganded datasets. For each unliganded ELIC5 cryo-EM dataset in a POPC, 3:1 POPC:DPOCL and 2:1:1 POPC:POPE:POPG lipid environment, snapshots of Loop C in the agonist binding site are shown from the 3DVA along the first variability component (i.e. reaction coordinate). For reference, the structural models of ELIC5 unliganded in 2:1:1 POPC:POPE:POPG (green) and ELIC5 agonist-bound in 2:1:1 POPC:POPE:POPG (blue) are shown. The cryo-EM maps show movement in Loop C in the ELIC5 unliganded PC dataset.

bioRxiv preprint doi: https://doi.org/10.1101/2025.10.07.680764; this version posted October 7, 2025. The copyright holder for this preprint (which was not certified by peer review) is the author/funder, who has granted bioRxiv a license to display the preprint in perpetuity. It is made available under aCC-BY 4.0 International license.

Supplementary Figure 7:

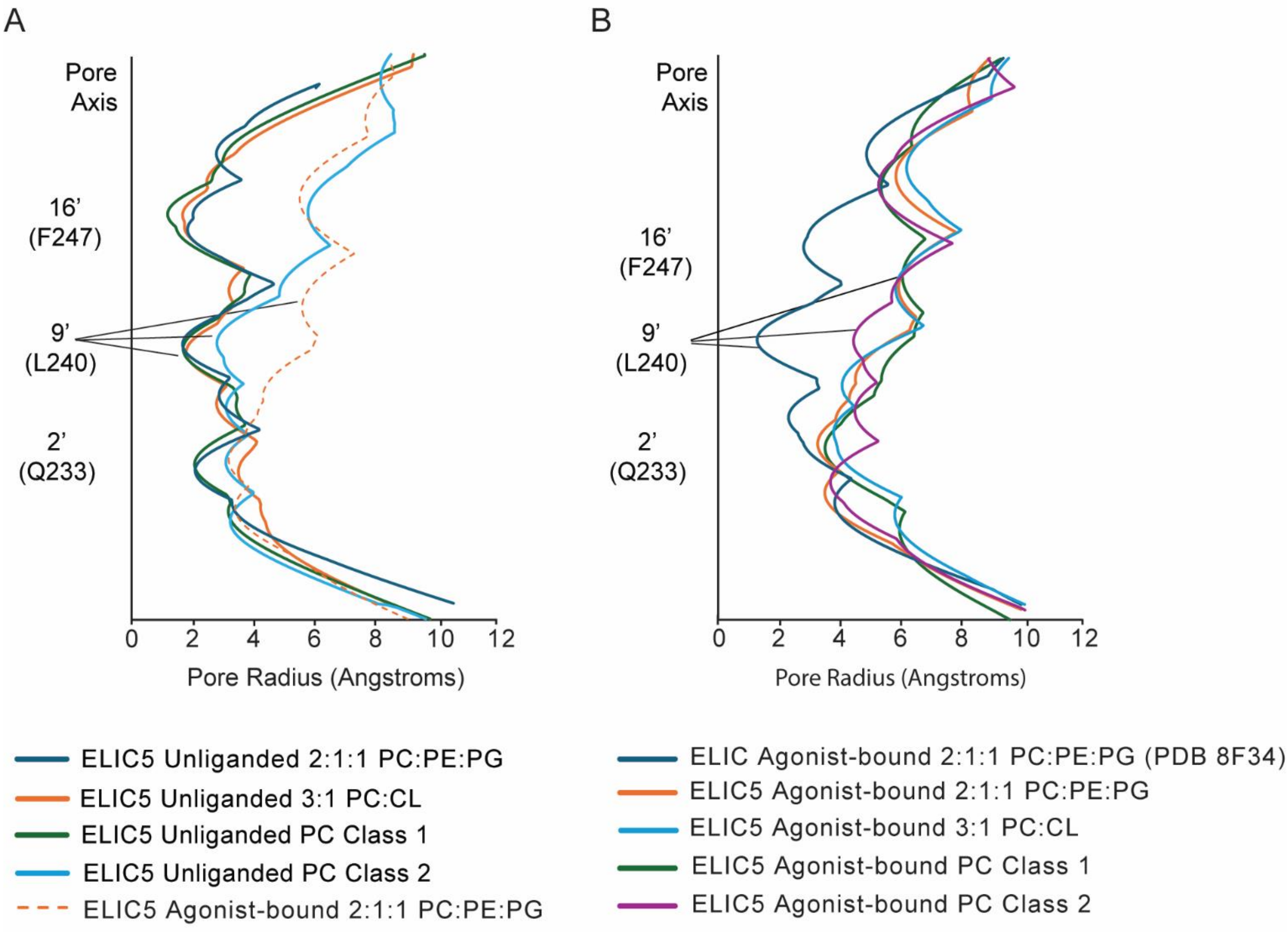

Supplementary Figure 7: Analysis of pore dimensions in the ELIC5 structures. Plot of pore radius for the indicated structures from this study generated using HOLE. The pore profiles in (A) are unliganded ELIC5 structures with the ELIC5 agonist-bound structure in 2:1:1 POPC:POPE:POPG for comparison. The pore profiles in (B) are the agonist-bound structures with agonist-bound WT ELIC (PDB 8F34) structures for comparison.

bioRxiv preprint doi: https://doi.org/10.1101/2025.10.07.680764; this version posted October 7, 2025. The copyright holder for this preprint (which was not certified by peer review) is the author/funder, who has granted bioRxiv a license to display the preprint in perpetuity. It is made available under aCC-BY 4.0 International license.

Supplementary Figure 8

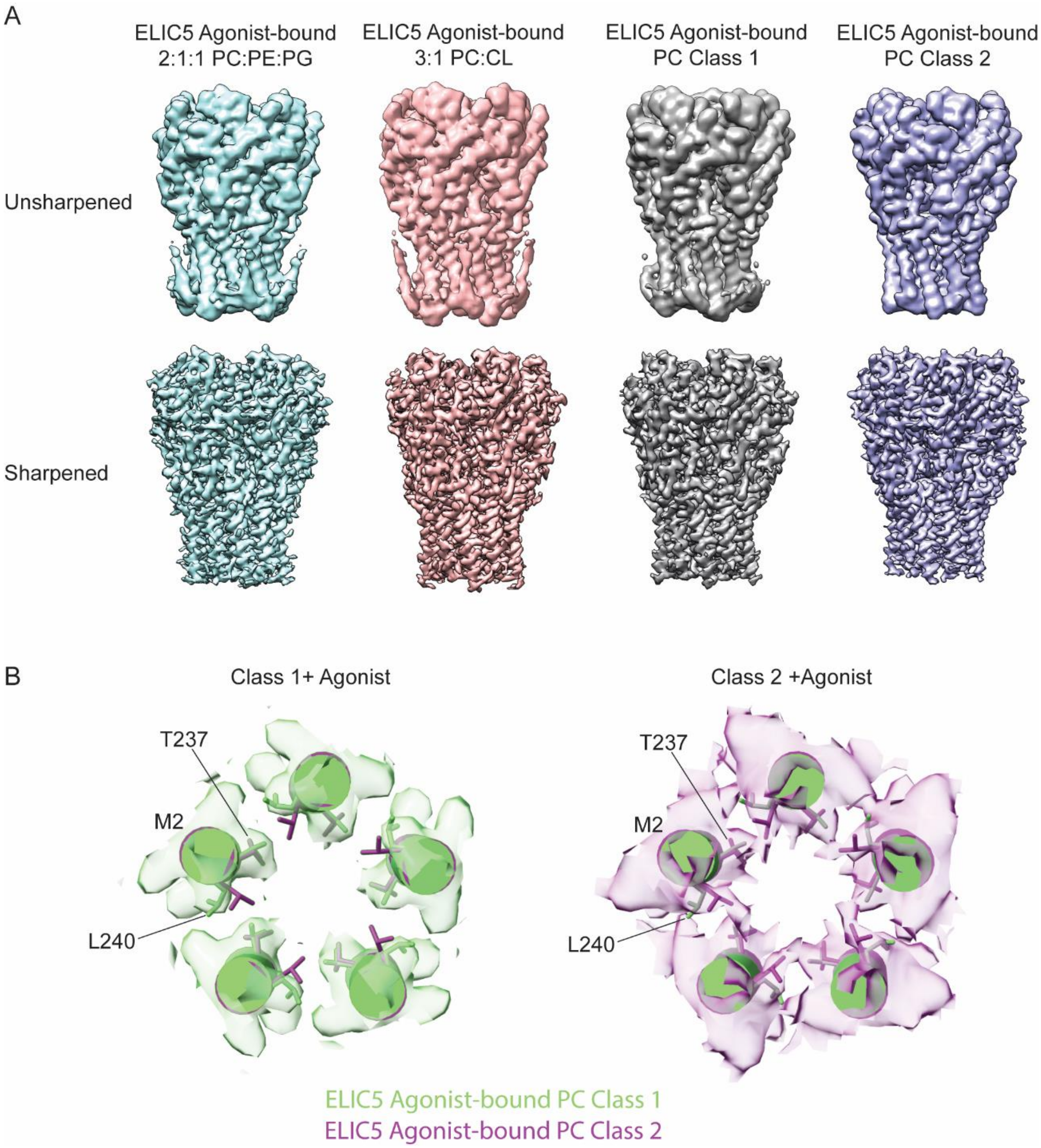

Supplementary Figure 8: Structural comparison of ELIC5 agonist-bound structures in different lipid environments. (A) Unsharpened and sharpened cryo-EM maps of ELIC5 with agonist in 2:1:1 POPC:POPE:POPG, 3:1 POPC:DPOCL, and POPC lipid environments. The cryo-EM density for M4 is absent in the agonist-bound POPC structures, and therefore this helix was not included in the models. (B) Comparison of “class 1 + agonist” (green) and “class 2 + agonist” (purple) cryo-EM maps for ELIC5 in POPC showing M2 with a view down the pore axis. The models of M2 for the corresponding structures are shown with stick representation of L240 and T237. The structures differ in the side chain orientation of L240.

bioRxiv preprint doi: https://doi.org/10.1101/2025.10.07.680764; this version posted October 7, 2025. The copyright holder for this preprint (which was not certified by peer review) is the author/funder, who has granted bioRxiv a license to display the preprint in perpetuity. It is made available under aCC-BY 4.0 International license.

Supplementary Figure 9:

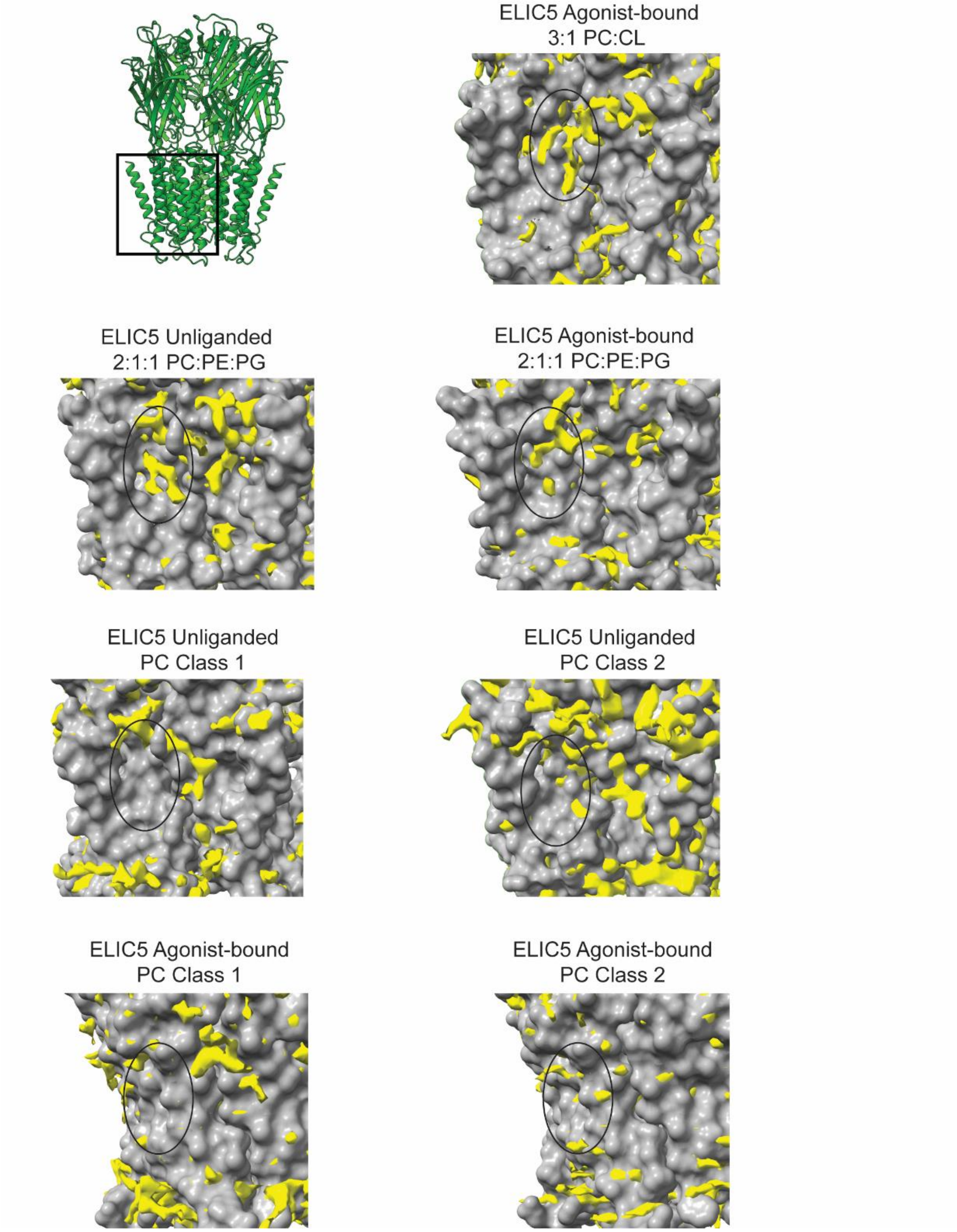

Supplementary Figure 9: Non-protein densities around the TMD of ELIC structures. For each indicated structure, a surface representation of the protein model is shown of the TMD centered on the M3-M4 groove (indicated by a black circle). The non-protein cryo-EM densities are shown in yellow using the same density threshold for all structures. Weak lipid-like densities are appreciated near M3 and M4 in the 2:1:1 POPC:POPE:POPG and 3:1 POPC:DPOCL structures with the clearest density observed in the unliganded 3:1 POPC:DPOCL structure. Note that M4 was not resolved in the agonist-bound POPC structures so this helix was not included in the models.

bioRxiv preprint doi: https://doi.org/10.1101/2025.10.07.680764; this version posted October 7, 2025. The copyright holder for this preprint (which was not certified by peer review) is the author/funder, who has granted bioRxiv a license to display the preprint in perpetuity. It is made available under aCC-BY 4.0 International license.

Supplementary Figure 10:

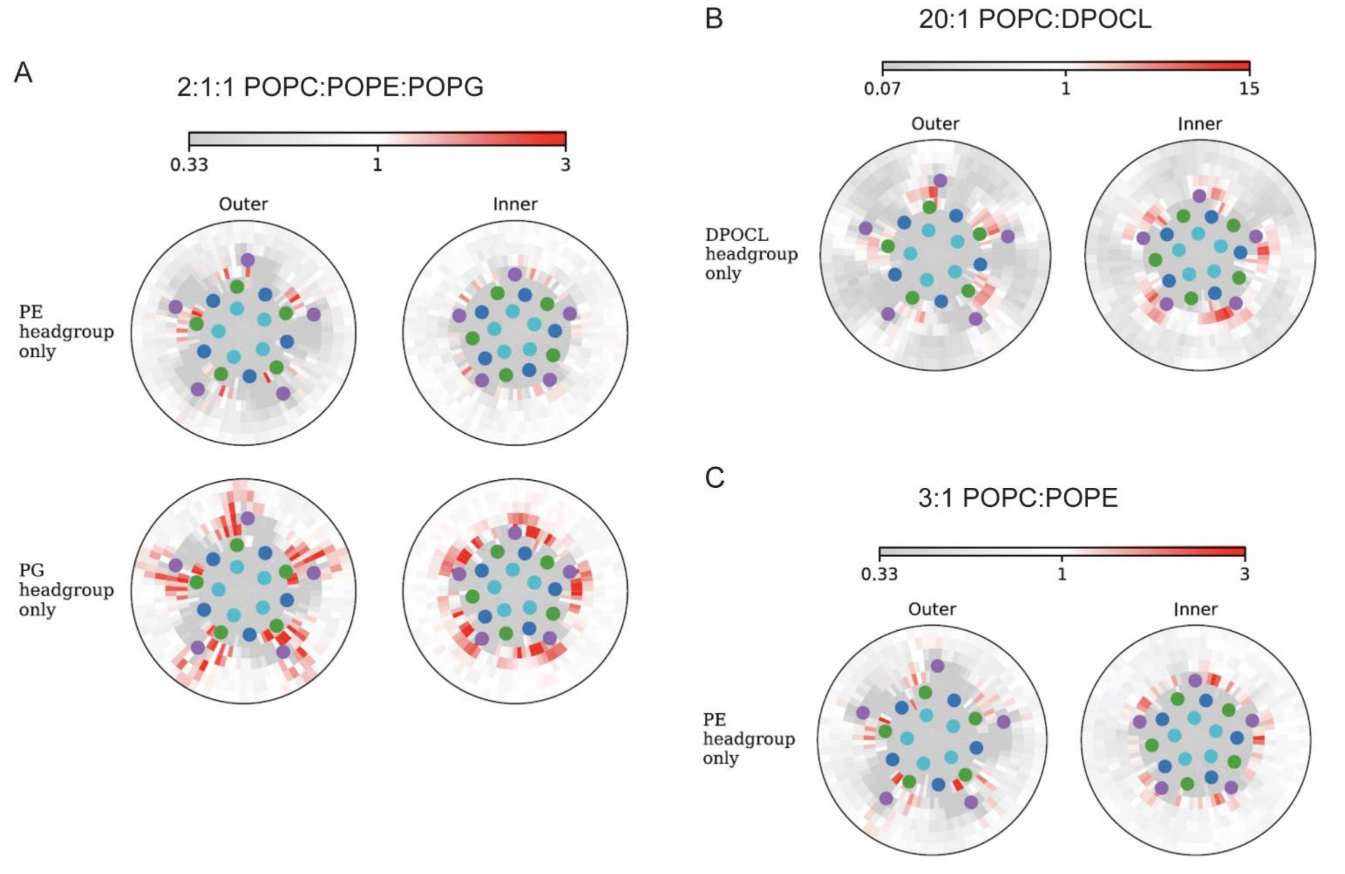

Supplementary Figure 10: Phospholipid interactions with the agonist-bound ELIC5 open-channel structure (PDB 9NGC). Radial distribution plots from the CGMD simulation of the ELIC5 open-channel structure in the three indicated lipid environments: (A) 2:1:1 POPC:POPE:POPG in which POPE and POPG density enrichment is shown, (B) 20:1 POPC:DPOCL in which DPOCL density enrichment is shown, and (C) 3:1 POPC:POPE in which POPE density enrichment is shown within 5 nm of the protein center. The analysis was performed with the phospholipid headgroups only. The colored dots represent the center of mass of each TMD helix. Shown are the outer and inner leaflet.

bioRxiv preprint doi: https://doi.org/10.1101/2025.10.07.680764; this version posted October 7, 2025. The copyright holder for this preprint (which was not certified by peer review) is the author/funder, who has granted bioRxiv a license to display the preprint in perpetuity. It is made available under aCC-BY 4.0 International license.

Supplementary Figure 11:

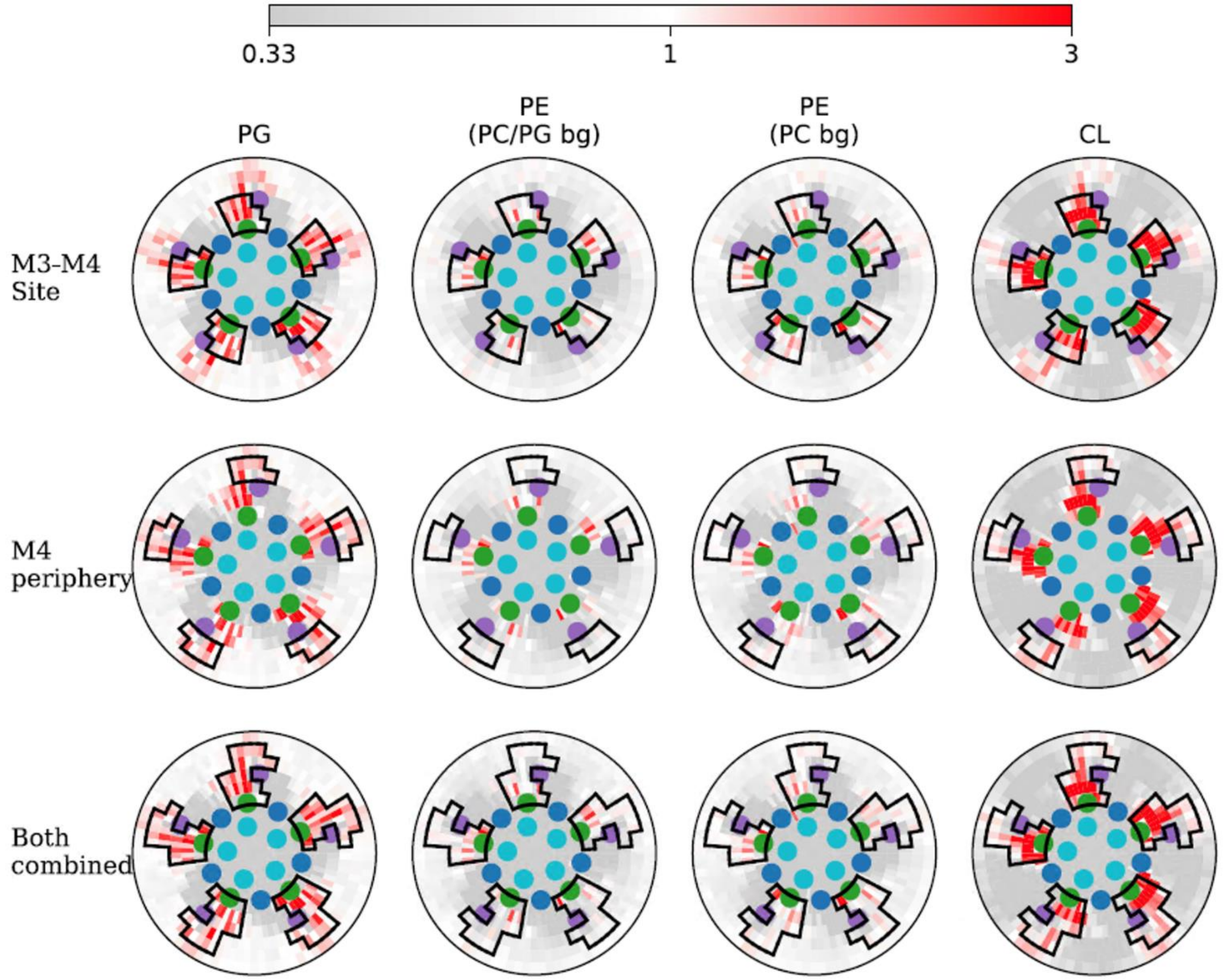

	PG (2:1:1)	PE (2:1:1)	PE (3:1)	CL (20:1)
**Outer M3-M4**	-1.4 ± 0.2	-0.6 ± 0.1	-0.6 ± 0.2	-1.3 ± 0.1
**Outer M4 periphery**	-0.5 ± 0.1	0.1 ± 0.1	0.1 ± 0.1	-0.1 ± 0.1
**Both Combined**	-1.4 ± 0.1	-0.3 ± 0.1	-0.4 ± 0.1	-0.9 ± 0.1

Supplementary Figure 11: Affinity of phospholipids for the agonist-bound ELIC5 open-channel structure (PDB 9NGC). Radial distribution plots as shown in Supplementary Figure 10 for the outer leaflet. “PG” is the distribution for POPG from a 2:1:1 POPC:POPE:POPG membrane. “PE (PC/PG bg)” is the distribution for POPE from a 2:1:1 POPC:POPE:POPG membrane. “PE (PC bg)” is the distribution for POPE from a 3:1 POPC:POPE membrane. “CL” is the distribution for DPOCL from a 3:1 POPC:DPOCL membrane. The black outlines demarcate the site definitions for the M3-M4 outer leaflet site, an M4 peripheral site, or both combined. The table shows ΔG_{bind} values for the indicated lipids at these sites (± SD).

bioRxiv preprint doi: https://doi.org/10.1101/2025.10.07.680764; this version posted October 7, 2025. The copyright holder for this preprint (which was not certified by peer review) is the author/funder, who has granted bioRxiv a license to display the preprint in perpetuity. It is made available under aCC-BY 4.0 International license.

Supplementary Figure 12:

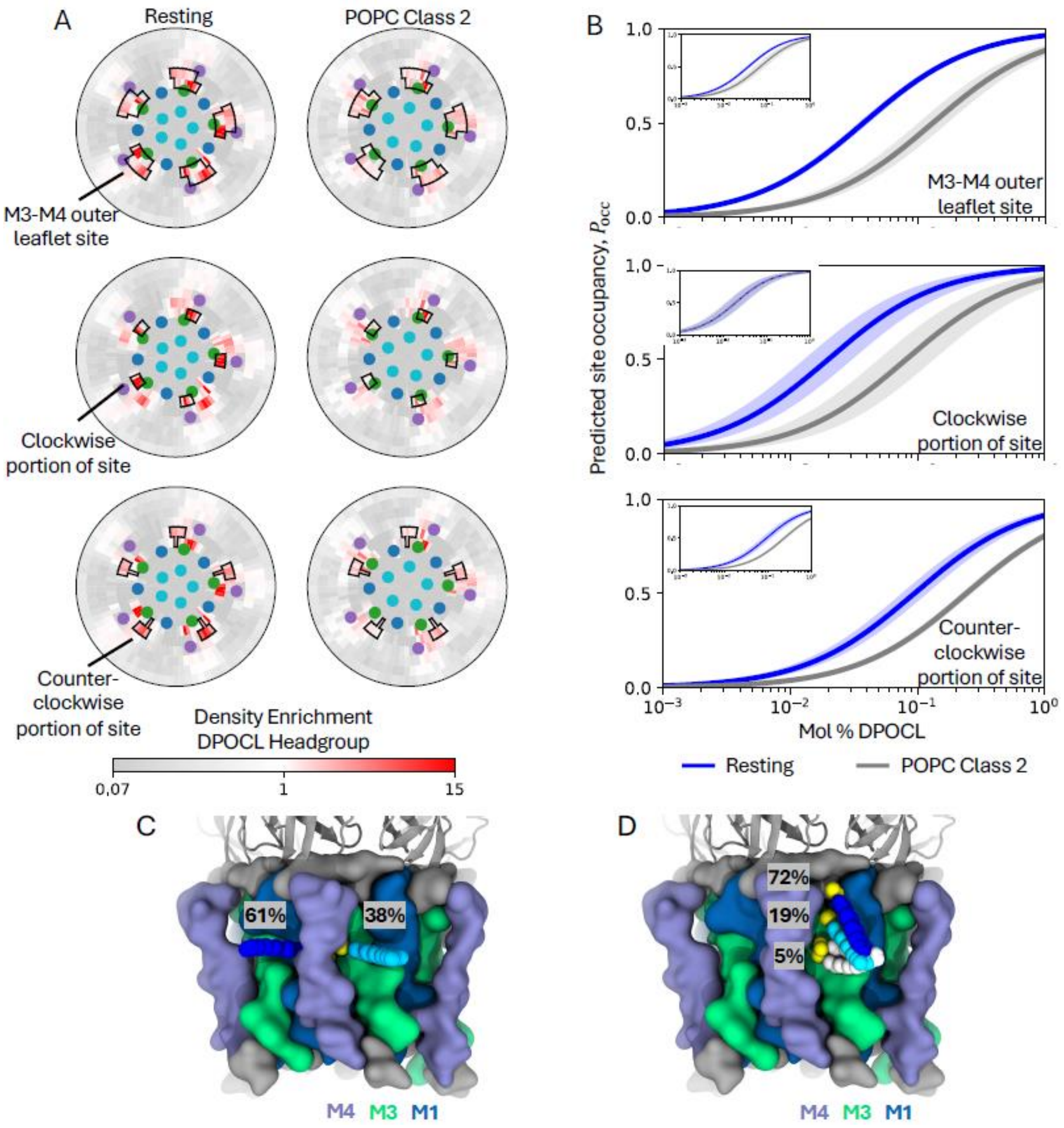

Supplementary Figure 12: State-dependent binding of DPOCL to an outer leaflet M3-M4 site. (A) Radial distribution plots from the CGMD simulation showing density enrichment of DPOCL headgroups in the outer leaflet within 5 nm of the protein center. The colored dots represent the center of mass of each TMD helix. Black outlines demarcate the site definitions for the M3-M4 outer leaflet site (top), the clockwise (CW) portion of the site (middle), and the counterclockwise (CCW) portion of the site (bottom). (B) Predicted occupancy (P_{occ}) of the M3-M4 site (top), CW portion (middle) and CCW portion (bottom), as a function of mol% DPOCL. The shaded region represents 95% confidence interval with n=20 for the

bioRxiv preprint doi: https://doi.org/10.1101/2025.10.07.680764; this version posted October 7, 2025. The copyright holder for this preprint (which was not certified by peer review) is the author/funder, who has granted bioRxiv a license to display the preprint in perpetuity. It is made available under aCC-BY 4.0 International license.

resting state and n=15 for POPC class 2, which is the “class 2 unliganded” structure. Insets show P_{occ} when POPC Class 2 site ΔG_{bind} values are corrected for accessible area. (C-D) Molecular image of the resting state ELIC5 structure from the CGMD simulation with the average positions of most occupied clusters of DPOCL when its headgroup is in the CW portion (C) and CCW portion (D) of the site near M3-M4. The average DPOCL headgroup positions for each cluster are shown as yellow VdW spheres; average tail positions are shown as blue or white VdWspheres. The percentages correspond to the relative frequency of each binding mode.

bioRxiv preprint doi: https://doi.org/10.1101/2025.10.07.680764; this version posted October 7, 2025. The copyright holder for this preprint (which was not certified by peer review) is the author/funder, who has granted bioRxiv a license to display the preprint in perpetuity. It is made available under aCC-BY 4.0 International license.

Supplementary Figure 13:

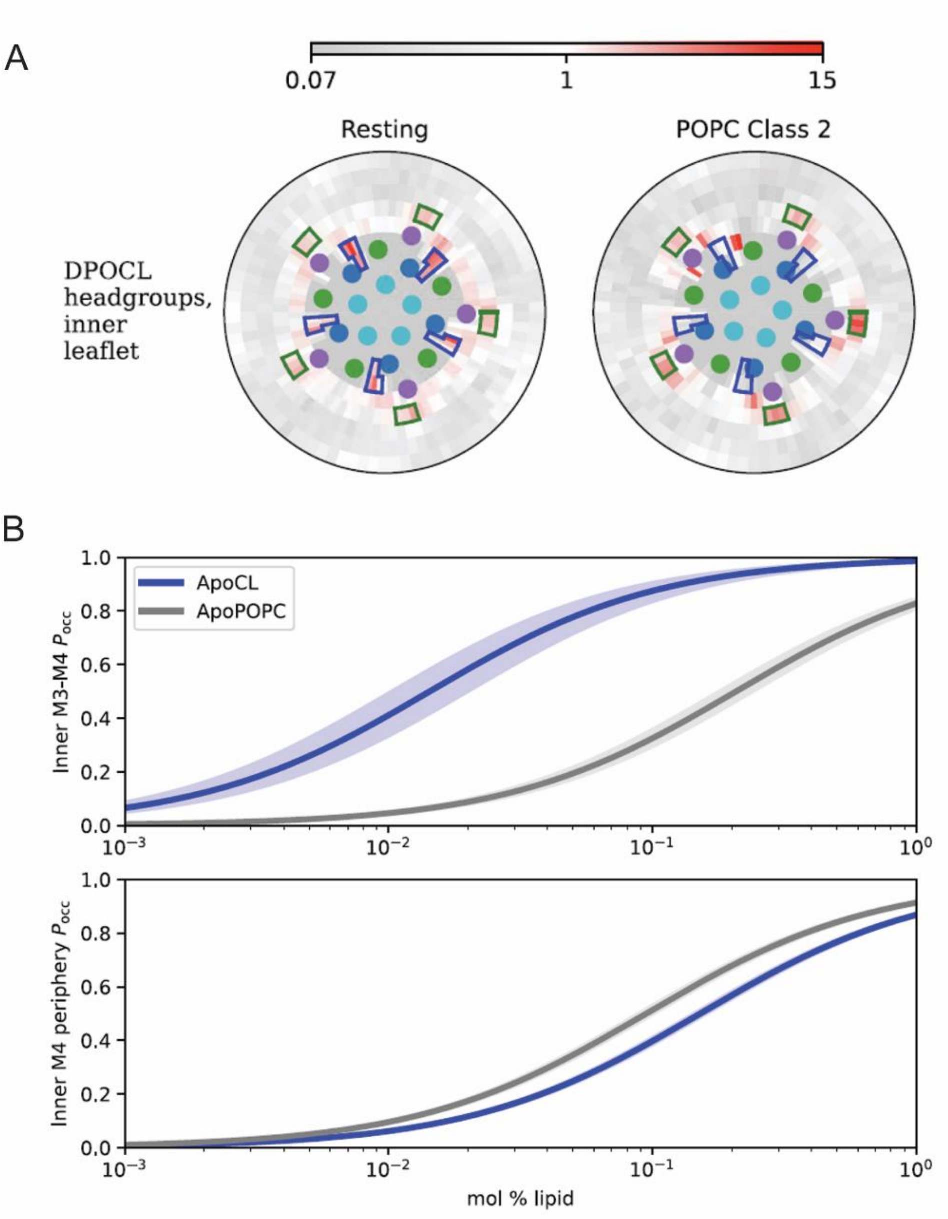

C

	Resting	POPC Class 2	POPC Class 2 (area corrected)
**Outer M3-M4**	-2.1 ± 0.1	-1.5 ± 0.3	-1.7 ± 0.3
M3-M4 CW portion	-2.5 ± 0.6	-1.6 ± 0.6	-2.5 ± 0.6
M3-M4 CCW portion	-1.5 ± 0.3	-0.9 ± 0.1	-0.9 ± 0.1
**Inner M1-M4**	-2.7 ± 0.5	-1.0 ± 0.2	-1.2 ± 0.2
**Inner M4 periphery**	-1.2 ± 0.1	-1.5 ± 0.1	-1.5 ± 0.1

Supplementary Figure 13: State-dependent binding of DPOCL to an inner leaflet M1-M4 site. (A) Radial distribution plots from the CGMD simulation showing density enrichment of DPOCL headgroups in the inner leaflet within 5 nm of the protein center. The colored dots represent the center of mass of each TMD helix. The outlines demarcate the site definitions for the M1-M4 inner leaflet site and an M4 periphery site

bioRxiv preprint doi: https://doi.org/10.1101/2025.10.07.680764; this version posted October 7, 2025. The copyright holder for this preprint (which was not certified by peer review) is the author/funder, who has granted bioRxiv a license to display the preprint in perpetuity. It is made available under aCC-BY 4.0 International license.

in the inner leaflet. (B) Predicted occupancy (P_{occ}) of the M1-M4 site (top) and the M4 periphery site (bottom) as a function of mol% DPOCL. The shaded region represents 95% confidence interval with n=20 for the resting state and n=15 for POPC class 2, which is the "class 2 unliganded" structure. (C) Table shows ΔG_{bind} values for DPOCL at all the sites from this figure and Supplementary Figure 12, comparing the resting state structure and POPC class 2, which is the "unliganded class 2" structure (± SD). Also indicated are the ΔG_{bind} values for POPC class 2 when P_{occ} is corrected for accessible surface area.

bioRxiv preprint doi: https://doi.org/10.1101/2025.10.07.680764; this version posted October 7, 2025. The copyright holder for this preprint (which was not certified by peer review) is the author/funder, who has granted bioRxiv a license to display the preprint in perpetuity. It is made available under aCC-BY 4.0 International license.

Supplementary Table 1: Summary of cryo-EM data collection and refinement parameters

	ELIC5 in spNW15 nanodiscs with 2:1:1 POPC:POPE:POPG EMD-72875 PBD 9YF3	ELIC5 with propylamine in spNW15 nanodiscs with 2:1:1 POPC:POPE:POPG EMD-72859 PBD 9YEQ	ELIC5 in spNW15 nanodiscs with 3:1 POPC:DPOCL EMD-72856 PBD 9YEN
**Data collection and processing**			
Magnification	120,000	120,000	120,000
Voltage 9kV	200	200	200
Electron Exposure	50.09	50.09	50.09
Defocus Range	-1 to -2.4	-1 to -2.4	-1 to -2.4
Pixel Size	1.184	1.184	1.184
Symmetry Imposed	C5	C5	C5
Initial Particle Images (no)	373,058	726,368	920,554
Final Particle Images (no)	33,194	34,036	44,494
Map Resolution (A)	3.37	3.33	3.28
FSC Threshold	0.143	0.143	0.173
REFINEMENT			
Initial Model Used	PDB 8VUW	PDB 8VUW	PDB 8VUW
Model Resolution (A)	3.4	3.4	3.3
FSC Threshold	0.5	0.5	0.5
Map Sharpening B Factor (A)	-185.0	-183.4	-181.6
MODEL COMPOSITION			
Non-hydrogen Atoms	12700	12,770	12,700
Protein Residues	1550	1550	1550
Ligands	0	5	5
B FACTORS			
Protein	8.59	30.92	8.35
Ligand		20.00	20.00
RMS DEVIATIONS			
Bond lengths	0.005	0.005	0.005
Bond Angles	1.077	1.076	1.159
VALIDATION			
MolProbity Score	1.92	1.57	1.91
Clashscore	13	4.55	14.86
Poor Rotamers (%)	0	0	0
RAMACHANDRAN PLOT			
Favored (%)	95.78	95.13	96.43
Allowed (%)	4.22	4.87	3.57
Disallowed (%)	0	0	0

bioRxiv preprint doi: https://doi.org/10.1101/2025.10.07.680764; this version posted October 7, 2025. The copyright holder for this preprint (which was not certified by peer review) is the author/funder, who has granted bioRxiv a license to display the preprint in perpetuity. It is made available under aCC-BY 4.0 International license.

	ELIC5 with propylamine in spNW15 nanodiscs with 3:1 POPC:DPOCL EMD-72858 PBD 9YEP	ELIC5 in spNW15 nanodiscs with POPC Class 1 EMD-72861 PBD 9YES	ELIC5 in spNW15 nanodiscs with POPC Class 2 EMD-72865 PBD 9YEW
**Data collection and processing**			
Magnification	120,000	120,000	20,000
Voltage 9kV	200	200	200
Electron Exposure	50.09	50.09	50.09
Defocus Range	-1 to -2.4	-1 to -2.4	-1 to -2.4
Pixel Size	1.184	1.184	1.184
Symmetry Imposed	C5	C5	C5
Initial Particle Images (no)	1,684,189	323,426	323,426
Final Particle Images (no)	48,171	21,891	8,937
Map Resolution (A)	3.18	3.65	3.76
FSC Threshold	0.143	0.143	0.143
REFINEMENT			
Initial Model Used	PDB 8VUW	PDB 8VUW	PDB 8VUW
Model Resolution (A)	3.5	3.9	4.2
FSC Threshold	0.5	0.5	0.5
Map Sharpening B Factor (A)	-177.8	-192.5	-177.1
MODEL COMPOSITION			
Non-hydrogen Atoms	12,700	12,700	12,700
Protein Residues	1,550	1,550	1,550
Ligands	0	0	0
B FACTORS			
Protein	85.34	88.88	124.48
Ligand	0	0	0
RMS DEVIATIONS			
Bond lengths	0.005	0.005	0.008
Bond Angles	1.108	1.152	1.431
VALIDATION			
MolProbity Score	1.81	1.95	2.25
Clashscore	11.34	14.99	20.73
Poor Rotamers (%)	0	0	0
RAMACHANDRAN PLOT			
Favored (%)	96.43	96.10	93.18
Allowed (%)	3.57	3.90	6.82
Disallowed (%)	0.00	0.00	0.00

bioRxiv preprint doi: https://doi.org/10.1101/2025.10.07.680764; this version posted October 7, 2025. The copyright holder for this preprint (which was not certified by peer review) is the author/funder, who has granted bioRxiv a license to display the preprint in perpetuity. It is made available under aCC-BY 4.0 International license.

	ELIC5 with propylamine in spNW15 nanodiscs with POPC Class 1 EMD-72867 PBD 9YEY	ELIC5 with propylamine in spNW15 nanodiscs with POPC Class 2 EMD-72869 PBD 9YF1	
**Data collection and processing**			
Magnification	120,000	120,000	
Voltage 9kV	200	200	
Electron Exposure	50.09	50.09	
Defocus Range	-1 to -2.4	-1 to -2.4	
Pixel Size	1.184	1.184	
Symmetry Imposed	C5	C5	
Initial Particle Images (no)	358,356	358,356	
Final Particle Images (no)	48,730	48,449	
Map Resolution (A)	3.43	3.28	
FSC Threshold	0.143	0.143	
REFINEMENT			
Initial Model Used	PDB 8VUW	PDB 8VUW	
Model Resolution (A)	3.7	3.4	
FSC Threshold	0.5	0.5	
Map Sharpening B Factor (A)	-204.2	-186.9	
MODEL COMPOSITION			
Non-hydrogen Atoms	11,505	11,505	
Protein Residues	1,390	1,390	
Ligands	5	5	
B FACTORS			
Protein	78.30	71.73	
Ligand	20.00	20.00	
RMS DEVIATIONS			
Bond lengths	0.004	0.005	
Bond Angles	0.928	1.056	
VALIDATION			
MolProbity Score	2.27	1.81	
Clashscore	27.64	8.33	
Poor Rotamers (%)	0		
RAMACHANDRAN PLOT			
Favored (%)	95.06	94.78	
Allowed (%)	4.94	5.22	
Disallowed (%)	0.00	0.00	

bioRxiv preprint doi: https://doi.org/10.1101/2025.10.07.680764; this version posted October 7, 2025. The copyright holder for this preprint (which was not certified by peer review) is the author/funder, who has granted bioRxiv a license to display the preprint in perpetuity. It is made available under aCC-BY 4.0 International license.

Supplementary Video 1: The 3DVA reaction coordinate along the first variability component for the unliganded ELIC5 structure in POPC nanodiscs. The video shows a side view of the full ion channel.

Supplementary Video 2: The 3DVA reaction coordinate along the first variability component for the unliganded ELIC5 structure in POPC nanodiscs. The video shows a top-down view along the pore axis of the ion channel from the extracellular side.
